# The Canonical Approach at High Temperature Revisited

**DOI:** 10.3390/e28101164

**Published:** 2026-10-21

**Authors:** Kouji Kashiwa, Hiroaki Kouno

**Affiliations:** 1 Department of Computer Science and Engineering, Fukuoka Institute of Technology, Wajiro, Fukuoka 811-0295, Japan; 2 Department of Physics, Saga University, Saga 840-8502, Japan

**Keywords:** quantum chromodynamics, canonical approach, imaginary chemical potential

## Abstract

This study examines an apparent discrepancy that can arise between the canonical construction and the Taylor expansion constructed from a high-temperature thermodynamic potential restricted to the trivial Polyakov-loop sector in the presence of the Roberge–Weiss transition. We show that the discrepancy originates from the distinction between the full Roberge–Weiss-periodic partition function and a representation restricted to a single Polyakov-loop sector. We further discuss how this distinction is affected by the thermodynamic limit and why it does not cause a problem for the standard finite-volume canonical formulation used in lattice QCD simulations. We also discuss how to connect the full RW-periodic partition function with a single-sector expression in the thermodynamic limit.

## 1. Introduction

Exploring the non-perturbative properties of quantum chromodynamics (QCD) at finite temperature (*T*) and finite real quark chemical potential ($\mu_{R}$) is crucial for understanding our universe; see, e.g., Ref. [1]. At finite *T* and $\mu_{R}=0$, lattice QCD simulations are feasible; these non-perturbative properties can, in principle, be investigated rigorously. However, at finite $\mu_{R}$, QCD suffers from the sign problem. Consequently, various methods are required to circumvent this issue; see, e.g., Refs. [2,3].

To mitigate the sign problem, several methods have been proposed so far; prominent examples include the reweighting method [4,5], the Taylor expansion method [6,7], the canonical approach [8,9,10], and the analytic continuation method [11]. Unfortunately, these methods are restricted to the $\mu_{R}/T<1$ region. To overcome these limitations, some other approaches based on complexifying dynamical variables have recently attracted significant attention, such as the Lefschetz thimble method [12,13,14], the complex Langevin method [15,16], and the path optimization method [17,18,19]. However, these techniques are still under development and face their own challenges. In this work, we focus on the canonical approach.

It is well known that the imaginary chemical potential ($\mu_{I}$) region is directly related to the physical real chemical potential region; see, e.g., Refs. [2,20]. Specifically, we can construct the grand canonical partition function at finite $\mu =(\mu_{R},0)$ from that at finite $\mu =(0,\mu_{I})$ [8]. This procedure forms the basis for the canonical approach [21,22,23,24,25,26]. In this framework, we can construct the canonical partition function with a fixed quark number using the grand canonical partition function at finite $\mu_{I}$. Consequently, the imaginary chemical potential region serves as a testing ground for examining the validity of analytical methods. In this paper, we discuss an apparent discrepancy between the canonical construction based on the full RW-periodic partition function and the Taylor expansion based on a sector-restricted high-temperature thermodynamic potential. We show that this discrepancy becomes relevant in the presence of the RW transition and originates from the distinction between the full RW-periodic partition function and its representation within a single Polyakov-loop sector.

At finite $\mu_{I}$, the system exhibits unique properties, including Roberge–Weiss (RW) periodicity, the RW transition, and the RW endpoint:

**RW periodicity** At finite $\mu_{I}$, thermodynamic quantities and order parameters exhibit a nontrivial 2π/3 periodicity along the θ-axis, where $\theta \equiv \mu_{I}/T$ [8]. This is generally known as RW periodicity.
**RW transition** The origin of the RW periodicity differs between the low- and high-temperature regions. This distinction arises from the balance between gluon and quark contributions to the grand canonical partition function ($Z_{\mathrm{GC}}$). At θ=(2k−1)π/3 with k∈Z, several quantities exhibit singularities at high *T* [8]. Quantities that are odd and even with respect to θ show first- and second-order singularities, respectively. These singularities characterize a phase transition known as the RW transition.
**RW endpoint** Although thermodynamic quantities oscillate smoothly along the θ-axis at low *T*, they develop singularities at high *T*, as explained above. Due to this change in behavior, the first-order RW transition line must terminate at an endpoint. This is called the RW endpoint, and its corresponding temperature is denoted as the RW endpoint temperature, $T_{\mathrm{RW}}$. The order of the phase transition at this endpoint depends on the quark masses; see, e.g., Refs. [27,28,29,30].
**$Z_{3}$ images** The regions separated by the RW transition at high *T* correspond to distinct $Z_{3}$ images. These are characterized by one trivial Polyakov-loop sector, where the phase is approximately 0, and two nontrivial Polyakov-loop sectors, where the phases are approximately 2π/3 and 4π/3.

**Figure 1:**
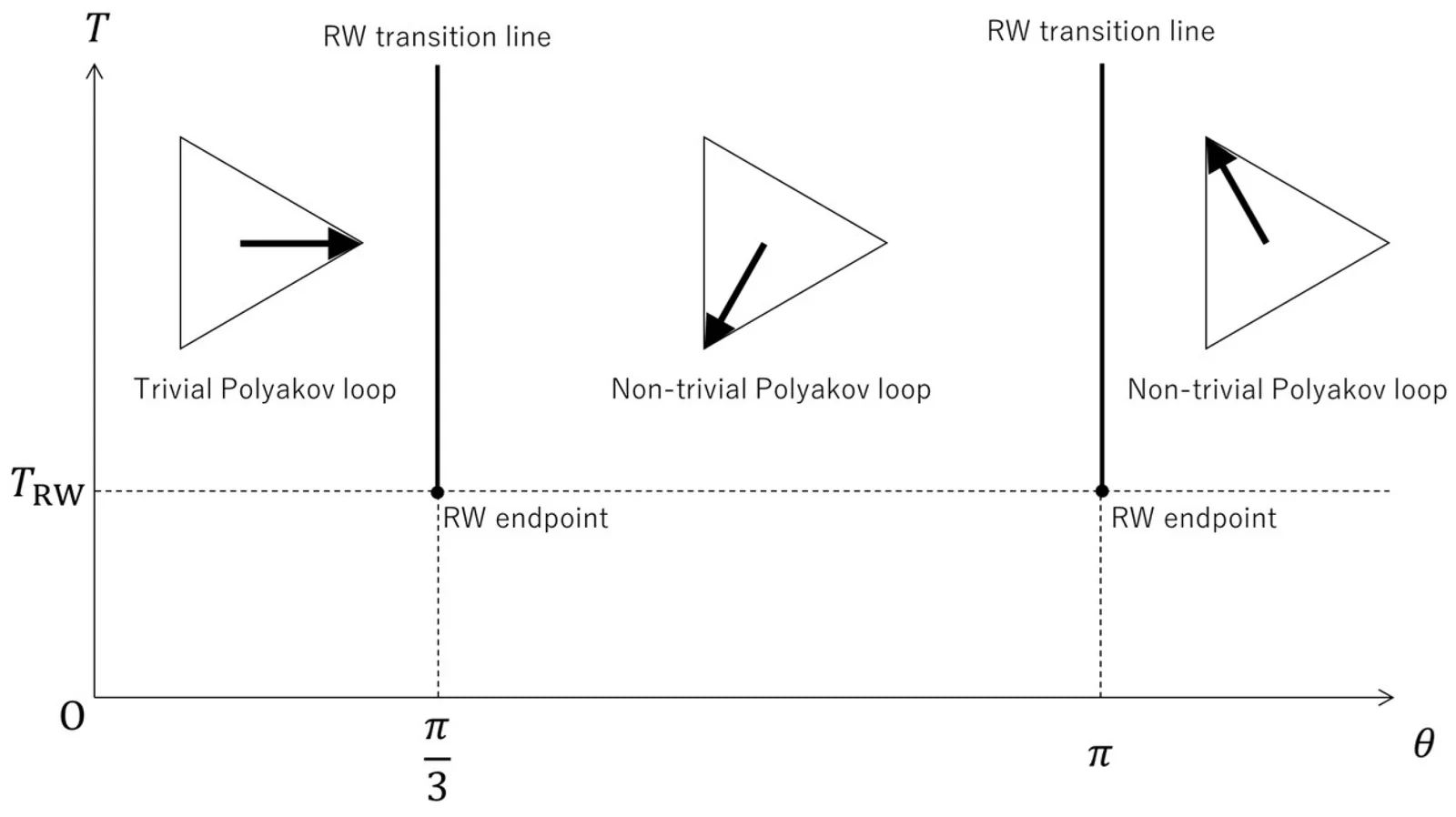
The schematic figure of the QCD phase diagram in the *T*-θ plane. The vertical solid lines indicate the RW transition lines above the RW endpoint temperature $T_{\mathrm{RW}}$. The arrows indicate the phase of the Polyakov loop in the trivial and nontrivial $Z_{3}$ sectors.

For a more detailed discussion of the imaginary chemical potential, see, e.g., Ref. [20]. The schematic figure is shown in Figure 1.

The canonical approach is based on the Fourier transformation and the fugacity expansion. It has been widely used to investigate the finite-$\mu_{R}$ region in lattice QCD simulations and QCD effective models. The RW periodicity strongly affects this construction, as discussed in Section 2. In this study, we examine an apparent discrepancy between the canonical construction based on the full RW-periodic partition function and the Taylor expansion with a high-temperature representation restricted to a single Polyakov-loop sector. It should be noted that the high-temperature region has recently attracted significant attention due to newly proposed insights, such as the potential existence of exotic phases; see, e.g., Refs. [31,32]. These recent developments further motivate our present study. This paper is organized as follows. In the next section, we explain the properties of QCD in the $\mu_{I}$ region. Section 2 introduces the canonical approach and its associated paradox. Section 3 presents a detailed discussion of the paradox. We also discuss how to connect the full RW periodic partition function with a single-sector representation in the thermodynamic limit. Finally, Section 4 is devoted to a summary of this research.

## 2. Canonical Approach

It is well known that the canonical partition function ($Z_{C}$) for a fixed quark number (*Q*) can be constructed from the grand canonical partition function ($Z_{\mathrm{GC}}$) evaluated at finite θ. The relationship is given by

(1) $$\begin{array}{cc} Z_{C}\left(Q\right)\; & =\sum_{n=-\infty }^{\infty }\langle n|e^{-\beta \hat{H}}\delta_{n,Q}|n\rangle =\frac{1}{2\pi }\int_{-\pi }^{\pi }e^{iQ\theta }Z_{\mathrm{GC}}\left(\theta \right)\;d\theta , \end{array}$$

where $\hat{H}$ denotes the Hamiltonian, n∈Z represents the eigenvalues of the quark number operator $\hat{N}$, and Q∈Z. This derivation is based on the Fourier representation of the Kronecker delta. This formulation has already been widely applied to both lattice QCD simulations [21,22,23,24,25,26] and QCD effective model calculations [33,34,35,36,37]; for recent progress, see, e.g., Ref. [38]. As expected, the resulting partition function describes the canonical ensemble, enabling the evaluation of various physical quantities.

Using the fugacity expansion, the grand canonical partition function at finite $\mu_{R}$ can be expressed in terms of the canonical partition functions as

(2) $$\begin{array}{cc} Z_{\mathrm{GC}}\left(\mu_{R}\right)\; & =\sum_{Q=-\infty }^{\infty }\exp\left(Q\frac{\mu_{R}}{T}\right)Z_{C}\left(Q\right)=\sum_{Q=-\infty }^{\infty }\xi^{Q}Z_{C}\left(Q\right), \end{array}$$

where ξ is the fugacity, defined as

(3) $$\begin{array}{cc} \xi & =\exp\left(\frac{\mu_{R}}{T}\right). \end{array}$$

In the canonical approach, we usually focus on the grand canonical partition function and related quantities; however, the canonical partition function itself is also of great physical interest. Specifically, canonical partition functions are directly related to the multiplicity distributions measured in collision experiments; for experimental data, see Refs. [39,40], and for a recent review, see Ref. [41]. Indeed, this connection has been utilized to combine lattice QCD results with experimental data at finite density via the analysis of Lee–Yang zeros [42,43]; see, e.g., Ref. [44].

The canonical partition function (1) for a fixed quark number *Q* can be constructed using $Z_{\mathrm{GC}}$ at finite *T* and θ as

(4) $$\begin{array}{cc} Z_{C}\left(Q\right)\; & =\frac{1+z^{Q}+z^{2Q}}{2\pi }\int_{-\pi /3}^{\pi /3}e^{iQ\theta }Z_{\mathrm{GC}}\left(\theta \right)\;d\theta \\ & \left\{ \begin{array}{cc} =\begin{array}{c} \frac{3}{2\pi }\int_{-\pi /3}^{\pi /3}e^{iQ\theta }Z_{\mathrm{GC}}\left(\theta \right)\;d\theta \; \end{array} & \left(Q=3k\right)\\ 0 & \left(Q\neq 3k\right) \end{array},\right. \end{array}$$

where k∈Z and *z* is the $Z_{3}$ factor defined as

(5) $$\begin{array}{cc} z & =\exp\left(\frac{2\pi i}{3}\right). \end{array}$$

In this study, we consider the three-color system, and thus the center symmetry is $Z_{3}$. The property shown in Equation (4) is also valid for almost all quantities except the Polyakov loop. This exception is known as the Polyakov-loop paradox [45], which was resolved by using the modified Polyakov loop in Ref. [46]. In the following, we do not explicitly include *T* as an argument in the partition function, as we focus on the μ-dependence at a fixed *T*.

Using the fugacity expansion, the grand canonical partition function can be expressed as

(6) $$\begin{array}{cc} Z_{\mathrm{GC}}\left(\mu_{R}\right)\; & =\sum_{k=-\infty }^{\infty }\exp\left(3k\frac{\mu_{R}}{T}\right)Z_{C}\left(3k\right)=\sum_{k=-\infty }^{\infty }\exp\left(Q^{\prime }\frac{\mu_{R}}{T}\right)Z_{C}\left(Q^{\prime }\right), \end{array}$$

where $Q^{\prime }=3k$. Here, we use the well-known fact that only quark numbers that are multiples of $N_{c}$ (with $N_{c}=3$) contribute to $Z_{C}$ due to RW periodicity, as shown in Equation (4); see also Ref. [8]. In the above procedure, we start from $Z_{\mathrm{GC}}\left(\theta \right)$ to obtain $Z_{C}\left(Q\right)$, but this relation can also be inverted.

At high *T*, physical observables such as partition function, entropy, and number density should exhibit smooth oscillation at finite θ within a specific $Z_{N_{c}}$ image. The RW transition, characterized by a singularity, arises from the crossing between distinct images. Thus, for a single $Z_{N_{c}}$ image, we can approximate the form of $Z_{\mathrm{GC}}\left(\theta \right)$ as

(7) $$\begin{array}{cc} Z_{\mathrm{GC}}\left(T,\mu_{I}\right) & =\frac{1}{2}a_{0}+\sum_{l=1}^{l_{\max}}a_{l}\cos\left(l\theta \right), \end{array}$$

where $l_{\max}$ denotes the cutoff of the summation, which is essentially +∞, and $a_{l}\in R$ represents the Fourier coefficients; see Ref. [47] for a practical fitting example. This expression is simply a truncated Fourier series. If the oscillating behavior is sufficiently smooth, $l_{\max}$ can be truncated to a small integer. Although this functional form inherently possesses only the 2π periodicity, the full RW periodicity can be reproduced by considering the superposition of its $Z_{N_{c}}$ replicas. Performing the integration in Equation (1), only the terms with l=Q contribute to the canonical partition function due to the orthogonality of the Fourier basis. Taking into account the properties of the RW periodicity shown in Equation (4), we obtain the grand canonical partition function as

(8) $$\begin{array}{cc} Z_{\mathrm{GC}}\left(\mu_{R}\right)\; & =\frac{1}{2}\sum_{k=-l_{\max}/3}^{l_{\max}/3}\exp\left(3k\frac{\mu_{R}}{T}\right)a_{3k}=\frac{1}{2}\sum_{k=-l_{\max}/3}^{l_{\max}/3}\exp\left(Q^{\prime }\frac{\mu_{R}}{T}\right)a_{Q^{\prime }}, \end{array}$$

where $Q^{\prime }=3k$. This confirms that only contributions with $Q^{\prime }=3k$ survive in the sum.

In contrast, when employing analytic continuation or Taylor expansion, non-3k contributions can emerge. The grand potential Ω expressed via the Taylor expansion is given by

(9) $$\begin{array}{cc} \Omega (T,\mu_{R})\; & =c_{0}+c_{2}\mu_{R}^{2}+c_{4}\mu_{R}^{4}+\ldots , \end{array}$$

(10) $$\begin{array}{cc} \Omega (T,\mu_{I})\; & =c_{0}-c_{2}\mu_{I}^{2}+c_{4}\mu_{I}^{4}+\ldots , \end{array}$$

where $c_{n}\in R$ are the Taylor coefficients. By definition, the Taylor coefficients obtained on the real and imaginary chemical-potential axes are related by analytic continuation, provided that no singularity is encountered. It is important, however, to distinguish the order of the Taylor expansion from the Fourier-harmonic index and the canonical quark number. The RW selection rule constrains the Fourier harmonics and canonical sectors of the full RW-periodic partition function to indices that are multiples of three. It does not imply that the Taylor expansion contains only powers whose orders are multiples of three. A single-sector high-temperature expression may contain Fourier harmonics with indices not divisible by three, and these harmonics contribute to its Taylor coefficients around θ=0. To discuss these Taylor coefficients in a model-independent manner, we examine both the perturbative one-loop calculation and the strong-coupling expansion in Section 2.1 and Section 2.2, respectively.

### 2.1. High Temperature Region Above RW Endpoint

The perturbative one-loop effective potential, evaluated via a semi-classical expansion of the gluon field in the Polyakov gauge, is expressed as

(11) $$\begin{array}{cc} \Omega (T,\mu_{I})\; & =\frac{2N_{f}T^{2}}{\pi^{2}}\sum_{c=1}^{3}\sum_{n=1}^{\infty }\frac{m^{2}K_{2}\left(\frac{nm}{T}\right)}{n^{2}}\cos\left(2\pi nx\right), \end{array}$$

here *g* is the gauge coupling constant, *m* represents the current quark mass, $N_{f}$ is the number of flavors, and $K_{2}\left(\cdot \right)$ denotes the modified Bessel function of the second kind. The variable $A_{4}^{c}$ is the diagonal (semi-)classical part of the temporal gluon field $A_{4}$ with color index *c*, and its value is determined by minimizing Ω. The dimensionless variable *x* is defined as

(12) $$\begin{array}{cc} \; & x=\frac{gA_{4}^{c}+\theta +\pi }{2\pi }. \end{array}$$

The additional π originates from the anti-periodic temporal boundary condition for quarks. Although the imaginary chemical potential is not explicitly considered in Ref. [48], the dimensionless variable θ can be regarded as a background U(1) phase in the fermionic temporal boundary condition. It therefore enters the one-loop effective potential through the same phase combination as the temporal gauge-field background, as shown in Equation (12). Accordingly, the formulation of Ref. [48] can be extended to finite imaginary chemical potential by replacing the gauge-field phase with the corresponding combination including θ. At sufficiently high temperatures *T*, the quantity $gA_{4}^{c}$ takes the values 0, $\frac{2\pi }{3}$, or $\frac{4\pi }{3}$ depending on θ, ensuring that the absolute value of the Polyakov loop is 1. These values correspond precisely to the $Z_{3}$ images. In other words, the trivial $Z_{3}$ image characterizes the trivial Polyakov-loop sector, while the nontrivial images characterize the nontrivial sectors.

In the chiral limit, in the absence of spontaneous chiral symmetry breaking, Equation (11) simplifies to

(13) $$\begin{array}{cc} \Omega (T,\mu_{I}) & =-\frac{4N_{f}\pi^{2}T^{4}}{3}B_{4}\left(x\right)=-\frac{4N_{f}\pi^{2}T^{4}}{3}\left(x^{4}-2x^{3}+x^{2}-\frac{1}{30}\right). \end{array}$$

This result is derived using the small-argument expansion of the modified Bessel function

(14) $$\begin{array}{cc} K_{2}\left(z\right) & =\frac{2}{z^{2}}-\frac{1}{2}+\ldots , \end{array}$$

along with the Fourier series representation of the fourth-order Bernoulli polynomial $B_{4}\left(x\right)$, given by

(15) $$\begin{array}{cc} B_{4}\left(x\right) & =-\frac{3}{\pi^{4}}\sum_{n=1}^{\infty }\frac{\cos(2\pi nx)}{n^{4}}. \end{array}$$

From Equations (11) and (13), it is evident that contributions with n≠3k are present in this regime. Consequently, the Taylor coefficients in Equation (10) must inherently include these n≠3k terms. In contrast, such n≠3k contributions strictly vanish in the canonical approach due to the Roberge–Weiss (RW) transition and its associated periodicity, as demonstrated in Equation (8).

### 2.2. Low Temperature Region Below RW Endpoint

Qualitative properties of the low-temperature regime can be inferred from the strong-coupling limit, as it effectively describes the confined, chiral symmetry broken phase; see, e.g., Refs. [49,50]. The thermodynamic potential in this limit is given by

(16) $$\begin{array}{cc} \Omega (T,\mu_{I}) & ∼T\ln\left[\frac{1}{4}\cos\left(3\theta \right)\right]. \end{array}$$

This expression exclusively contains 3k contributions, which naturally follows from Roberge–Weiss (RW) periodicity in the absence of singularities, unlike in the high-*T* regime.

Higher-order terms in the strong-coupling expansion of the effective potential include combinations such as

(17) $$\begin{array}{c} \Phi e^{i\theta },\;\Phi e^{-2i\theta },\;\bar{\Phi }e^{-i\theta },\;\bar{\Phi }e^{2i\theta }, \end{array}$$

where Φ denotes the Polyakov loop, defined as

(18) $$\begin{array}{cc} \Phi & ={\mathrm{tr}}_{c}\left[P\exp\left(ig\oint d\tau A_{4}^{c}\right)\right], \end{array}$$

here P denotes path ordering, ${\mathrm{tr}}_{c}$ is the trace over color space, τ is imaginary time, and $\bar{\Phi }$ is the complex conjugate of the Polyakov loop. At first glance, the presence of these terms suggests the existence of non-3k contributions; see, e.g., Ref. [51]. However, these effectively reduce to 3k contributions because the terms in Equation (17) can be rewritten as

(19) $$\begin{array}{c} \Psi ,\;\Psi e^{-3i\theta },\;\bar{\Psi },\;\bar{\Psi }e^{3i\theta }, \end{array}$$

where $\Psi \equiv \Phi e^{i\theta }$ is the so-called modified Polyakov loop. This modified Polyakov loop maintains the RW periodicity under the extended $Z_{3}$ symmetry, which is defined by the following transformations [52]:

(20) $$\begin{array}{cc} \Phi & \to \Phi \exp\left(-\frac{2\pi ik}{3}\right),\\ \bar{\Phi } & \to \bar{\Phi }\exp\left(\frac{2\pi ik}{3}\right),\\ e^{\pm i\theta } & \to e^{\pm i\theta }\exp\left(\pm \frac{2\pi ik}{3}\right). \end{array}$$

The modified Polyakov loop Ψ is invariant under the extended $Z_{3}$ transformation, which is equivalent to the shift symmetry discussed in Refs. [53,54,55,56]; see also Ref. [46]. On the RW transition line, this extended $Z_{3}$ symmetry is broken by the degeneracy of the vacuum. Due to this effect, the RW periodicity becomes singular and non-3k contributions appear in the single sector.

Based on the preceding discussion, the full thermodynamic function in the low-*T* regime contains only Fourier harmonics with indices n=3k. Its Taylor coefficients around θ=0 are therefore determined entirely by these RW-compatible Fourier modes. Hence, there is no discrepancy between the full RW-periodic description and the canonical construction in this regime. Furthermore, while a first-order phase transition induced by chiral symmetry may occur in close proximity to the RW endpoint, it does not affect the RW periodicity. Therefore, such a transition is irrelevant to the present discussion.

## 3. Discussion

At low temperatures below the RW endpoint, the effective potential at finite θ consists exclusively of contributions of 3k due to RW periodicity in the absence of the RW transition. Conversely, at temperatures above the RW endpoint, the RW periodicity is maintained by the $Z_{3}$ images. The thermodynamic potential can contain arbitrary n≠3k contributions. These observations imply that, above the RW endpoint temperature, a canonical reconstruction based on the full RW-periodic partition function does not reproduce the result obtained from a single-sector high-temperature expression unless the distinction between the two objects is taken into account.

The apparent discrepancy between the full RW-periodic result and the single-sector representation can be understood from their different Fourier structures, as explained below. By applying the Fourier series and its inverse as utilized in the canonical approach, we obtain

(21) $$\begin{array}{cc} \; & Z_{\mathrm{GC}}\left(\theta \right)∼\frac{1}{2\pi }\sum_{Q=-Q_{\max}}^{Q_{\max}}\left[\left\{\int_{-\pi }^{\pi }e^{iQ\theta^{\prime }}Z_{\mathrm{GC}}\left(\theta^{\prime }\right)\;d\theta^{\prime }\right\}e^{-iQ\theta }\right], \end{array}$$

where $Q_{\max}$ denotes the truncation cutoff for the Fourier sum, which is required by practical numerical evaluations.

Provided there is no information loss in Equation (21), the right-hand side rigorously equals the left-hand side, successfully reproducing $Z_{\mathrm{GC}}\left(\theta \right)$. According to Dirichlet’s theorem, this equality is naturally guaranteed in the limit $Q_{\max}\to \infty$ if $Z_{\mathrm{GC}}\left(\theta \right)$ is a smooth, continuous and 2π/3-periodic function. Crucially, even if $Z_{\mathrm{GC}}\left(\theta \right)$ exhibits a cusp (a discontinuity in its derivative), the series in Equation (22) still converges to the exact result as $Q_{\max}\to \infty$, as the Dirichlet conditions are sufficiently satisfied.

When there is a gap, the Gibbs phenomenon [57] occurs. Oscillatory overshoots emerge near the discontinuity. The relative magnitude of the overshoot does not vanish as $Q_{\max}$ increases, although the affected region becomes narrower. Instead, in the infinite-$Q_{\max}$ limit, the Fourier series converges merely to the arithmetic mean of the left- and right-hand limits at the gap. Therefore, while a gap induces persistent truncation artifacts, a cusp can be perfectly reconstructed via the Fourier representation.

In the Taylor expansion method, the thermodynamic quantities are expanded around μ=0 and matched with the results obtained via analytic continuation from $\mu_{I}$ to $\mu_{R}$. This procedure implies that, at high temperatures, the Taylor coefficients encode information exclusively from the trivial Polyakov-loop sector. Consequently, one must restrict the canonical approach to this trivial sector as well. Under this condition, the Fourier series in Equation (1) is modified to

(22) $$\begin{array}{cc} Z_{C}\left(Q\right) & =\frac{1}{2\pi }\left[\int_{-\pi }^{\pi }e^{iQ\theta }Z_{\mathrm{GC}}^{\mathrm{fix}}\left(\theta \right)\;d\theta \right], \end{array}$$

where $Z_{\mathrm{GC}}^{\mathrm{fix}}\left(\theta \right)$ is the grand canonical partition function restricted to the trivial Polyakov-loop sector, resulting in a Fourier series with a periodicity of 2π. Although imposing this restriction is generally challenging, it is straightforward in the perturbative one-loop calculation, as it simply requires setting $gA_{4}^{c}=0$.

Although the full RW-periodic reconstruction in Equation (21) does not reproduce the single-sector result at high $T>T_{\mathrm{RW}}$, Equation (22) does reproduce it because the sector-restricted function retains the Fourier harmonics with indices not divisible by three. This can be explicitly demonstrated by applying the Fourier decomposition from Equation (7) to the trivial Polyakov-loop sector with $m_{\max}\to \infty$:

(23) $$\begin{array}{cc} \frac{1}{2\pi }\int_{-\pi }^{\pi }d\theta \;e^{iQ\theta }\left[\frac{a_{0}}{2}+\sum_{m=1}^{\infty }a_{m}\cos\left(m\theta \right)\right] & =\left\{\begin{array}{cc} \frac{a_{0}}{2}, & Q=0,\\ \frac{a_{\left|Q\right|}}{2}, & Q\neq 0. \end{array}\right. \end{array}$$

yielding

(24) $$\begin{array}{cc} Z_{\mathrm{GC}}\left(T,\mu_{R}\right) & =\frac{a_{0}}{2}+\frac{1}{2}\sum_{Q=1}^{\infty }a_{Q}\left(\xi^{Q}+\xi^{-Q}\right)\underset{\mu_{R}=0}{⟶}\frac{1}{2}a_{0}+\sum_{Q=1}^{\infty }a_{Q}. \end{array}$$

This outcome matches precisely the exact result in Equation (7) for θ=0. These mathematical properties explain the apparent discrepancy discussed in this paper.

As discussed above, at low temperatures below $T_{\mathrm{RW}}$, the original $Z_{\mathrm{GC}}\left(\theta \right)$ is a continuous 2π/3-periodic function. Therefore, it naturally excludes n≠3k contributions. Therefore, both Equations (1) and (22) yield identical results in this regime. Finally, one might be concerned that restricting the Polyakov-loop sector could compromise the Lee–Yang zero analysis [42,43] for QCD. Indeed, the standard Lee–Yang zero analysis requires specific modifications within this framework, which are detailed in Appendix A.

In lattice QCD simulations, calculations are inherently restricted to finite volumes, precluding the appearance of strict thermodynamic singularities such as the RW transition. Physically, this mathematical distinction is strictly governed by the volume of the system. In finite-volume systems, thermodynamic singularities are inherently smeared. Consequently, any severe jump discontinuity (gap) is softened to a continuous or smooth behavior. This smearing effectively circumvents the Gibbs phenomenon, guaranteeing that the Fourier series converges to the exact result as dictated by Dirichlet’s theorem. Instead, information regarding the transition must be extracted by extrapolating to the thermodynamic limit, a standard practice in lattice computations. Consequently, the canonical approach remains valid for lattice QCD simulations since they operate within a finite-volume regime. However, if the infinite-volume limit is taken from the outset, at first glance, it seems that there is a difference between the full RW-periodic canonical reconstruction and the single-sector-restricted expression. To avoid this inconsistency, all of the three Polyakov-loop sectors are treated consistently. In Appendix B, we discuss how to connect the full RW periodic partition function with its single-sector counterpart in the thermodynamic limit. Another promising remedy involves coarse-graining via gradient flow [58,59,60]. Under flow evolution, the distribution of local (non-averaged) Polyakov loops clusters around each $Z_{3}$ domain in the deconfined phase, as recently demonstrated in lattice simulations of the three-dimensional Potts model and quenched QCD [61]. Through this procedure, it may be possible to systematically isolate the trivial $Z_{3}$ image, thereby validating the approach even in the thermodynamic limit.

## 4. Summary

In this study, we have revisited the canonical approach at high temperatures, specifically in the regime above the Roberge–Weiss (RW) endpoint temperature ($T_{\mathrm{RW}}$) at finite imaginary chemical potential ($\mu_{I}$). Our primary focus has been to assess the validity of the canonical approach in the presence of the RW transition.

In the low-temperature regime ($T<T_{\mathrm{RW}}$), where the RW transition is absent at finite $\theta =\mu_{I}/T$, all θ-even observables, such as the chiral condensate and entropy, are smooth and continuous functions. Consequently, the canonical approach remains strictly valid; the representational capacity of the 2π-periodic Fourier series and its inverse transformation are entirely sufficient in this context. In contrast, in the high-temperature regime ($T>T_{\mathrm{RW}}$) where the RW transition emerges at a finite θ in the thermodynamic limit, a canonical reconstruction based on the full RW-periodic partition function does not reproduce a high-temperature expression restricted to a single Polyakov-loop sector. The apparent discrepancy discussed in this paper is not a contradiction of the canonical formulation itself. It originates from comparing two different thermodynamic objects. One is the Fourier construction based on the full RW-periodic partition function. The other is a high-temperature expression restricted to a single Polyakov-loop sector. As discussed, this discrepancy inherently originates from the mathematical properties of the Fourier series representation.

The integration period of the Fourier series in the standard canonical approach encompasses both the trivial and nontrivial Polyakov-loop sectors, leading to a destructive cancellation. As a result, all terms disappear except those corresponding to multiples of three (the 3k contributions). If the physical quantities consist exclusively of 3k contributions, where k∈Z correspond to multiples of the quark number, the conventional canonical approach yields correct results. However, when non-3k contributions are physically relevant, it becomes necessary to explicitly restrict the gluon field to the trivial Polyakov-loop sector. This inadvertent cancellation between the trivial and nontrivial sectors lies at the heart of the paradox addressed in this paper.

In finite-volume systems, thermodynamic singularities such as the RW transition cannot occur. Consequently, the partition function remains smooth and continuous, maintaining RW periodicity and ensuring that only 3k contributions survive. This implies that the standard canonical construction remains well defined at finite volume, whereas care is required when it is combined with a sector-restricted expression defined directly in the thermodynamic limit. Therefore, one must exercise great caution when applying the canonical approach to QCD effective models, which typically assume the thermodynamic limit from the outset, unlike lattice QCD simulations. Naturally, the canonical approach remains robust even in the infinite-volume limit at low temperatures, where the RW transition does not occur.

Finally, we note another situation where a mismatch may arise. If a standard Feynman-diagrammatic treatment is used without a semi-classical gluon background, non-3k contributions can emerge even in the low-temperature regime. In that framework, neither the RW periodicity nor the RW transition is properly encoded [62]. This finding further underscores the crucial need for careful evaluation when pairing the canonical approach with QCD effective models.

This work therefore does not challenge the standard canonical formulation. Rather, it clarifies the interpretation of comparisons between the full RW-periodic partition function and sector-restricted high-temperature representations.

## Data Availability

No new data were created or analyzed in this study. Data sharing is not applicable to this article.

## Appendix A. Lee–Yang Zeros

By adopting the Fourier series presented in Equation (22), the RW transition can be partially reproduced from the perspective of the Lee–Yang zero analysis. Although the following discussion closely parallels that of Ref. [63], a key distinction is that the effective potential exhibits a 2π periodicity due to the explicit restriction of the Polyakov-loop sector to the trivial one.

In the high-*T* regime, the grand canonical partition function can be expressed as

(A1) $$\begin{array}{cc} Z_{\mathrm{GC}}\left(\mu \right) & ∼\sum_{k=-\infty }^{\infty }\frac{1}{2\pi }\exp\left(-\frac{k^{2}}{4T^{3}V_{3}c_{2}}+\frac{\mu }{T}\right), \end{array}$$

where μ is the complex chemical potential, $c_{2}$ is the second-order coefficient in the Taylor expansion of the effective potential, and $V_{3}$ denotes the spatial volume. This expression is nothing but the Jacobi theta function, defined as

(A2) $$\begin{array}{cc} \vartheta (z,\tau ) & =\sum_{k=-\infty }^{\infty }\exp\left(\pi ik^{2}\tau +2\pi ikz\right), \end{array}$$

with the following parameter identifications:

(A3) $$\begin{array}{cc} 2\pi iz & =\frac{\mu }{T},\pi i\tau =-\frac{1}{4T^{3}V_{3}c_{2}}. \end{array}$$

It is well established that the zeros of this theta function are located at

(A4) $$\begin{array}{cc} z & =\frac{2k+1}{2}+\frac{2l+1}{2}\tau , \end{array}$$

where k,l∈Z. Consequently, the partition function in Equation (A1) exhibits Lee–Yang zeros at

(A5) $$\begin{array}{cc} \frac{\mu }{T} & =\left(2k+1\right)\pi i-\frac{2l+1}{4T^{3}V_{3}c_{2}}. \end{array}$$

Taking the thermodynamic limit ($V_{3}\to \infty$), we can directly read off the transition at θ=Im(μ)/T=π(mod2π). This demonstrates that the RW transition point is partially reproduced when the system is restricted to the trivial Polyakov-loop sector. To capture the remaining RW transition points at θ=π/3 and 5π/3, analogous restrictions are required for the corresponding nontrivial Polyakov-loop sectors. In finite-volume systems, the grand canonical partition function naturally remains a smooth, continuous, and 2π/3-periodic function of θ. Under these conditions, the paradox discussed in the main text does not arise; therefore, explicitly restricting the calculation to the trivial Polyakov-loop sector is entirely unnecessary.

## Appendix B. Comments on a Single-Sector Thermodynamic Function

Let us denote a thermodynamic function restricted to a single Polyakov-loop sector by f(θ). We assume that f(θ) is an even function with period 2π. It can therefore be expanded in a Fourier series as

(A6) $$\begin{array}{cc} f(\theta ) & =\sum_{l=0}^{\infty }a_{l}\cos\left(l\theta \right), \end{array}$$

where the coefficients $a_{l}$ are independent of θ. We next introduce the following even and periodic step function:

(A7) $$\begin{array}{cc} g(\theta ) & =\left\{\begin{array}{cc} 1 & \left(-\frac{\pi }{3}+2\pi k<\theta <\frac{\pi }{3}+2\pi k\right),\\ \frac{1}{2} & \left(\theta =-\frac{\pi }{3}+2\pi k,\;\frac{\pi }{3}+2\pi k\right),\\ 0 & \left(\mathrm{otherwise}\right), \end{array}\right. \end{array}$$

where *k* is an arbitrary integer and this function satisfies

(A8) $$\begin{array}{cc} g\left(\theta \right)+g\left(\theta +\frac{2\pi }{3}\right)+g\left(\theta -\frac{2\pi }{3}\right) & =1. \end{array}$$

Using the function (A7), we can define

(A9) $$\begin{array}{cc} \Omega (\theta ) & =f\left(\theta \right)g\left(\theta \right)+f\left(\theta +\frac{2\pi }{3}\right)g\left(\theta +\frac{2\pi }{3}\right)+f\left(\theta -\frac{2\pi }{3}\right)g\left(\theta -\frac{2\pi }{3}\right). \end{array}$$

The resulting function has a shorter RW period 2π/3. In the following, we refer to this procedure as RW periodicization and to g(θ) as the selection function.

Since g(θ) is also even and periodic, it can be expanded as

(A10) $$\begin{array}{cc} g(\theta ) & =\sum_{m=0}^{\infty }b_{m}\cos\left(m\theta \right), \end{array}$$

where the coefficients $b_{m}$ are independent of θ. Defining

(A11) $$\begin{array}{cc} F(\theta ) & \equiv f(\theta )g(\theta ), \end{array}$$

and using the trigonometric addition formula, we obtain

(A12) $$\begin{array}{cc} F(\theta ) & =\left(\sum_{l=0}^{\infty }a_{l}\cos\left(l\theta \right)\right)\left(\sum_{m=0}^{\infty }b_{m}\cos\left(m\theta \right)\right)\\ & =\sum_{l=0}^{\infty }\sum_{m=0}^{\infty }a_{l}b_{m}\cos\left(l\theta \right)\cos\left(m\theta \right)\\ & =\sum_{l=0}^{\infty }\sum_{m=0}^{\infty }\frac{a_{l}b_{m}}{2}\left\{\cos[(l+m)\theta ]+\cos[(l-m)\theta ]\right\}\\ & =\sum_{n=0}^{\infty }c_{n}\cos\left(n\theta \right), \end{array}$$

where $c_{n}$ are coefficients independent of θ. Equation (A9) can then be written as

(A13) $$\begin{array}{cc} \Omega (\theta ) & =F\left(\theta \right)+F\left(\theta +\frac{2\pi }{3}\right)+F\left(\theta -\frac{2\pi }{3}\right). \end{array}$$

For each Fourier mode, the sum over the three shifted functions gives

(A14) $$\begin{array}{cc} & \cos\left(n\theta \right)+\cos\left(n\theta +\frac{2n\pi }{3}\right)+\cos\left(n\theta -\frac{2n\pi }{3}\right)=\left\{\begin{array}{cc} 3\cos(n\theta ), & n=3k,\\ 0, & n\neq 3k, \end{array}\right. \end{array}$$

where *k* is a non-negative integer. Consequently,

(A15) $$\begin{array}{cc} \Omega (\theta ) & =3\sum_{k=0}^{\infty }c_{3k}\cos\left(3k\theta \right), \end{array}$$

which explicitly exhibits the RW periodicity. An important point is that the coefficients $c_{3k}$ generally contain not only the original coefficients $a_{3k}$, but also contributions originating from $a_{3k+1}$ and $a_{3k+2}$. Thus, although the function contains only Fourier modes compatible with RW periodicity, the information carried by the non-3k components of the original single-sector function is not simply discarded.

This property can be seen more explicitly by considering the Fourier series of the selection function. For the step function, we find

(A16) $$\begin{array}{cc} g(\theta ) & =\frac{1}{3}+\frac{\sqrt{3}}{\pi }\sum_{k=0}^{\infty }\left\{\frac{\cos[(6k+1)\theta ]}{6k+1}+\frac{\cos[(6k+2)\theta ]}{6k+2}\right.\\ & \left.\;-\frac{\cos[(6k+4)\theta ]}{6k+4}-\frac{\cos[(6k+5)\theta ]}{6k+5}\right\}. \end{array}$$

The first term is the average value of g(θ). In particular, apart from the constant term, the Fourier expansion contains no modes whose indices are multiples of three. Therefore, applying the RW periodicization to g(θ) itself eliminates all nonconstant Fourier modes:

(A17) $$\begin{array}{cc} g\left(\theta \right)+g\left(\theta +\frac{2\pi }{3}\right)+g\left(\theta -\frac{2\pi }{3}\right) & =1, \end{array}$$

which reproduces Equation (A8).

To make the role of the non-3k components more transparent, Ω(θ) can equivalently be expressed as

(A18) $$\begin{array}{cc} \Omega (\theta ) & =\sum_{j=0}^{\infty }a_{3j}\cos\left(3j\theta \right)+G\left(\theta \right)+G\left(\theta +\frac{2\pi }{3}\right)+G\left(\theta -\frac{2\pi }{3}\right), \end{array}$$

where

(A19) $$\begin{array}{cc} G(\theta ) & \equiv f\left(\theta \right)\left[g\left(\theta \right)-\frac{1}{3}\right]. \end{array}$$

Although G(θ) itself contains contributions from all Fourier coefficients of f(θ), including $a_{3j}$, its three shifted copies in Equation (A18) reorganize these contributions into an RW-periodic function. The first term in Equation (A18) explicitly reproduces the part of f(θ) that already possesses the 2π/3 periodicity. The remaining contribution contains the modifications induced by multiplication with the selection function. In particular, Fourier components of the original single-sector function with indices not divisible by three can contribute to the surviving 3k modes through mode mixing with the Fourier components of g(θ). Therefore, RW periodicization does not simply amount to discarding the non-3k Fourier coefficients of the original single-sector function.

Thus, the RW periodicization leaves unchanged those components of f(θ) that already possess RW periodicity. The remaining components are mixed with the selection function, and only the combinations compatible with the 2π/3 periodicity survive. Importantly, this operation does not necessarily eliminate all contributions originating from the non-3k coefficients. Through mode mixing with the selection function, such contributions can be incorporated into the surviving RW-periodic Fourier coefficients. Therefore, if an appropriate selection function can be identified, as in the example above, one can construct an RW-periodic thermodynamic function from a thermodynamic potential defined within a single Polyakov-loop sector. Conversely, by comparing the result of the canonical construction with that obtained from a single-sector expansion, it may also be possible to infer the corresponding selection function.
